# How many sites should an orthopedic trauma prospective multicenter trial have? A marginal analysis of the Major Extremity Trauma Research Consortium completed trials

**DOI:** 10.1186/s13063-024-07917-0

**Published:** 2024-02-05

**Authors:** Lauren Allen, Robert V. O’Toole, Michael J. Bosse, William T. Obremskey, Kristin R. Archer, Lisa K. Cannada, Jaimie Shores, Lisa M. Reider, Katherine P. Frey, Anthony R. Carlini, Elena D. Staguhn, Renan C. Castillo

**Affiliations:** 1 Department of Health Policy and Management, Johns Hopkins University Bloomberg School of Public Health, 415 North Washington Street, Baltimore, MD 21205 USA; 2 Department of Orthopaedics, University of Maryland School of Medicine, Baltimore, MD 21201 USA; 3 Atrium Health Carolinas Medical Center, Charlotte, NC 28204 USA; 4 Department of Orthopaedic Surgery, Vanderbilt University Medical Center, Nashville, TN 37232 USA; 5 Novant Health Orthopedic Fracture Clinic, Charlotte, NC 28211 USA; 6 School of Medicine, Johns Hopkins University, Baltimore, MD 21287 USA

**Keywords:** Multicenter trials, Research operations, Financial management

## Abstract

**Background:**

Multicenter trials in orthopedic trauma are costly, yet crucial to advance the science behind clinical care. The number of sites is a key cost determinant. Each site has a fixed overhead cost, so more sites cost more to the study. However, more sites can reduce total costs by shortening the study duration. We propose to determine the optimal number of sites based on known costs and predictable site enrollment.

**Methods:**

This retrospective marginal analysis utilized administrative and financial data from 12 trials completed by the Major Extremity Trauma Research Consortium. The studies varied in size, design, and clinical focus. Enrollment across the studies ranged from 1054 to 33 patients. Design ranged from an observational study with light data collection to a placebo-controlled, double-blinded, randomized controlled trial. Initial modeling identified the optimal number of sites for each study and sensitivity analyses determined the sensitivity of the model to variation in fixed overhead costs.

**Results:**

No study was optimized in terms of the number of participating sites. Excess sites ranged from 2 to 39. Excess costs associated with extra sites ranged from $17K to $330K with a median excess cost of $96K. Excess costs were, on average, 7% of the total study budget. Sensitivity analyses demonstrated that studies with higher overhead costs require more sites to complete the study as quickly as possible.

**Conclusions:**

Our data support that this model may be used by clinical researchers to achieve future study goals in a more cost-effective manner.

**Trial registration:**

Please see Table 1 for individual trial registration numbers and dates of registration.

**Supplementary Information:**

The online version contains supplementary material available at 10.1186/s13063-024-07917-0.

## Background

Multicenter clinical trials in orthopedic trauma are crucial to advance the science behind clinical care but are also complex and costly [1]. Despite the ongoing burden of injury and normal inflation rates, the orthopedic trauma research community is called upon to propose gold-standard studies which address the most critical questions while government funding for trials has leveled if not reduced [2]. Currently, there are no evidence-based approaches for the financial management of multicenter trials in an orthopedic trauma population.

To our knowledge, there are no resources in the clinical trials management literature that address the issue of determining how many sites to have in a government-sponsored multicenter clinical trial. Some studies have helped set expectations for trial performance among sites participating in multicenter trials [3], but most have not taken the total cost to the study into account. At least one study has shown that reducing the number of sites, among multiple other things, reduces total costs, but this was demonstrated in a single, high-cost, industry-sponsored trial where the study was well funded to begin with [4]. For the most part, the existing literature addresses site selection in the context of streamlining study startup processes [5, 6]. However, that literature focuses on how to select sites not how many sites to select [7–10]. Mature research networks with long-standing investigator relationships, where the pool of sites to draw from consists of those that have already invested in and contributed to the networks’ past studies, have the privilege of grappling with a different issue. These networks must determine how many sites are needed to achieve requisite sample sizes without wasting funds on excess sites.

While optimizing the number of sites will not solve the full puzzle of financial management best practices, it may reduce the likelihood that a multicenter trial will fall into two unfavorable circumstances. First, with too few sites, studies may not reach enrollment targets within the required timeframes and fail due to the inability to produce useful results [11]. Second, with too many sites, precious funding needed to reach scientific goals is wasted on negligible gains in the overall time to study completion. This study proposes a model for determining the optimal number of sites to have in a prospective multicenter trial. Our hypothesis is that the optimal number of sites can be determined based on study characteristics, known costs, and predictable site enrollment contributions.

## Methods

### Studies and sites

This study is a retrospective marginal analysis of studies conducted by the Major Extremity Trauma Research Consortium (METRC), an orthopedic trauma clinical trials consortium which has been in operation since 2009 [12]. METRC has sponsored more than 35 multicenter trials, each conducted within a large network of trauma centers located throughout the USA and Canada.

The proposed model uses METRC financial and enrollment data from 12 studies which have completed enrollment. In addition, it uses market-average single Institutional Review Board (IRB) costs which are newly relevant as the single IRB provision of the revised Common Rule took effect in January 2020 [13].

While the studies used for analysis are completed, they are phenotypically similar to more recently funded or proposed METRC studies, and the network of participating trauma centers is relatively constant. Thus, these studies provide the most appropriate and realistic inputs for the model. Table 1 provides the full and abbreviated names, primary objectives, total enrollments, and the number of sites that participated in the included studies.

**Table 1:** Major Extremity Trauma Research Consortium (METRC) study characteristics

Study name (abbreviation, registration number, registration date)	Primary objective	Number	Sites
Assessment of severe extremity wound bioburden at the time of definitive wound closure or coverage: correlation with subsequent post-closure deep wound infection (**Bioburden**, NCT01496014, 12/21/2011) [14].	To assess the extremity wound bioburden at the time of definitive wound closure or coverage and its correlation with subsequent deep wound infection	608	34
Improving recovery after orthopaedic trauma: cognitive-behavioral therapy based physical therapy (**CBPT**, NCT03335657, 11/8/2017) [15].	To test the efficacy of a phone-based cognitive-behavioral-based physical therapy (CBPT) program for managing pain in service members and civilians at risk for poor outcomes following lower-extremity trauma	636	9
A prospective randomized trial to assess fixation strategies for severe open tibia fractures: modern ring external fixator versus internal fixation (**FIXIT**, NCT01494519, 12/19/2011) [16].	To compare the outcomes associated with modern ring external fixators versus standard internal fixation techniques in treating severe open tibia shaft or metaphyseal fractures with or without a bone defect of any size	258	37
A multicenter prospective observational study of nerve repair and reconstruction associated with major extremity trauma (**NERVE**, NCT02718768, 3/24/2016) [17].	To capture the detailed information about the treatment and long-term outcomes of peripheral nerve injury (PNI) resulting from upper extremity trauma	250	17
Outcomes after severe distal tibia, ankle, and/or foot trauma: comparison of limb salvage versus transtibial amputation (**OUTLET**, NCT01606501, 5/25/2012) [18].	To compare the outcomes following limb salvage vs. amputation of a severe distal tibia, ankle, and/or foot trauma	651	27
Supplemental perioperative oxygen to reduce surgical site infection after high-energy fracture surgery (**OXYGEN**, NCT01798810, 2/26/2013) [19].	To assess the efficacy of supplemental perioperative oxygen in the prevention of surgical site infections	1173	29
Improving pain management and long-term outcomes following high-energy orthopaedic trauma (**PAIN**, NCT01789216, 2/12/2013) [20].	To compare the outcomes among patients randomized to three groups receiving standard pain management and either (1) oral and intravenous placebo, (2) NSAIDS (oral meloxicam and intravenous ketorolac) and oral placebo, or (3) oral pregabalin and intravenous placebo	450	25
A prospective randomized trial to assess oral versus intravenous antibiotics for treatment of postoperative wound infection after extremity fractures (**POvIV**, NCT01714596, 10/26/2012) [21].	To assess the efficacy of oral antibiotic therapy vs. intravenous antibiotics in the treatment of acute infection after fixation of fractures or fusion of joints	371	28
PhBMP-2 versus autograft for critical size tibial defects: a multicenter randomized trial (**pTOG**, NCT00853489, 3/2/2009) [22].	To compare rhBMP-2 vs. autograft for critical size tibial defects	33	17
Transtibial amputation outcomes study (**TAOS**, NCT01821976, 4/1/2013) [23].	To compare the outcomes for patients undergoing a transtibial amputation and receiving an end-bearing tibia-fibula synostosis vs. standard posterior flap procedure	381	28
Streamlining trauma research evaluation with advanced measurement (**STREAM**, NCT02079714, 3/6/2014) [24].	To evaluate the reliability, validity, and responsiveness of the NIH Patient Reported Outcomes Measurement Information System (PROMIS) Outcomes Tools	1054	52
Local antibiotic therapy to reduce infection after operative treatment of fractures at high risk of infection: a multicenter randomized controlled trial (**VANCO**, NCT02227446, 8/28/2014) [25].	To compare the efficacy of using local vancomycin powder in the prevention of surgical site infections	1000	35

Of the more than 70 sites that are part of the METRC network, 59 of them participated in one or more of the 12 included studies. All but two of the represented sites are level 1 trauma centers. A little more than half of the sites are publicly owned, and the same number have fellowship programs which accept between 1 and 5 trainees per year. While catchment areas and demography vary widely, all sites are in urban settings.

### Characterizing study volume and complexity

**Fig. 1:**
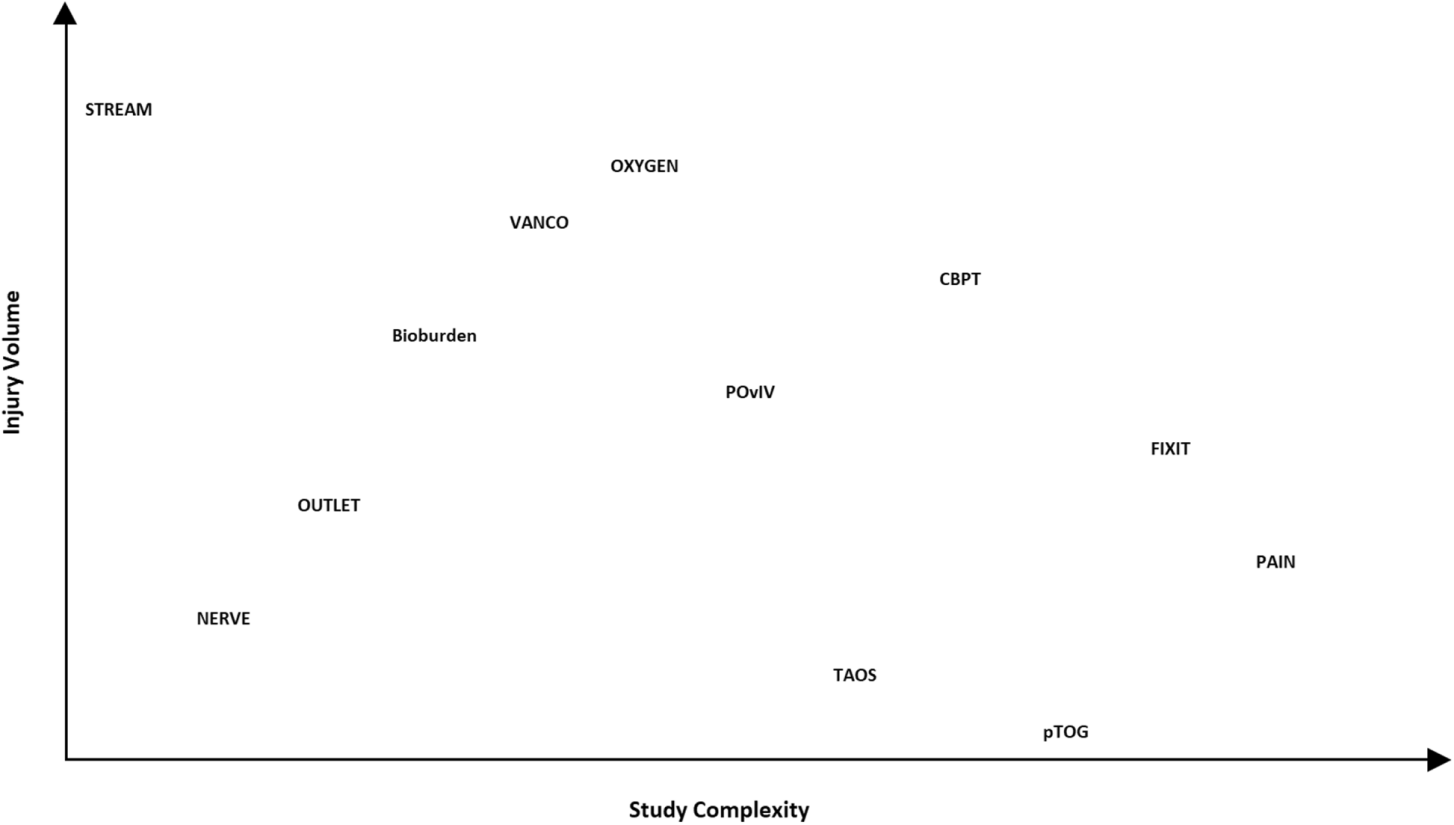
METRC study injury volume and study complexity*. *Full study names are listed in Table 1

The included studies all address key clinical questions in orthopedic trauma, but no two studies are exactly alike. Tables 2 and 3 along with Fig. 1 are intended to help place the studies along injury volume and study design complexity continua. High injury volume has historically motivated the inclusion of many participating sites, and study design complexity is one key driver of costs. Table 2 provides the principal inclusion criteria for each study along with an injury volume ranking (1–12, low to high) where the ranking is related to the restrictiveness of the inclusion criteria and overall volume of admissions for the study injury(s). Table 3 notes the design of each study and ranks them (1–12, low to high) according to the design and implementation complexity. These rankings were vetted for face validity by a group of five highly experienced clinical trialists. Figure 1 plots the studies according to the rankings in Tables 2 and 3 so that one can visualize how the studies relate to one another within these important parameters.

**Table 2:** Major Extremity Trauma Research Consortium study principal inclusion criteria and injury rarity rank

Study^a^	Principal inclusion criteria	Injury volume rank, *1–12, low to high*
pTOG	Open diaphyseal tibia fracture with bone defect ≥ 1 cm comprising > 50% circumference and treated with intramedullary nail.	1
TAOS	Early or delayed transtibial amputation.	2
NERVE	Peripheral nerve injuries (excl. purely sensory nerves) resulting from upper extremity trauma.	3
PAIN	Patients with isolated orthopedic trauma associated with moderate/high rates of chronic pain and nonunion, i.e., those with fractures to the ankle and midfoot, the tibia, the humerus, and the femur.	4
OUTLET	Selected open type III pilon and foot/ankle or severe open or closed crush/blast foot injuries.	5
FIXIT	Gustilo type IIIB and severe Gustilo type IIIA diaphyseal or metaphyseal tibia fractures.	6
POvIV	Patients with post-op wound infection following fractures or joint fusion of bone proximal to and including the tarsal/metatarsal joint or proximal to the carpal joints.	7
Bioburden	Open type III tibia fracture (plateau, shaft, and pilon) requiring additional procedure or below-knee amputation and delayed closure, skin grafting, and/or flap.	8
CBPT	Patients treated surgically for a high-energy lower-extremity injury at high risk for poor outcomes (presence of pre-defined psychosocial risk factors).	9
VANCO	High-energy tibial plateau and pilon fractures treated operatively with plate and screw fixation.	10
OXYGEN	High-energy tibial plateau, pilon, and calcaneous fractures treated operatively with plate and screw fixation.	11
STREAM	Patients enrolled in METRC’s FIXIT, OUTLET, OXYGEN, PAIN, and TAOS studies are eligible for co-enrollment into STREAM.	12

^a^Full study names are listed in Table 1

**Table 3:** Major Extremity Trauma Research Consortium study design and study complexity rank

Study^a^	Study design	Study complexity rank, *1–12, low to high*
STREAM	Observational study	1
NERVE	Observational study	2
OUTLET	Observational study with heavy data collection	3
Bioburden	Observational study with tissue sample collection and shipment	4
VANCO	Unblinded pragmatic trial	5
OXYGEN	Blinded pragmatic trial	6
POvIV	Explanatory trial; randomization to medication	7
TAOS	Explanatory trial; randomization to standard surgical technique	8
CBPT	Explanatory trial; randomization to new psychosocial intervention	9
pTOG	Explanatory trial; randomization to surgical technique and a device	10
FIXIT	Explanatory trial; randomization to surgical technique and a device	11
PAIN	Explanatory trial; double blinded, placebo controlled	12

^a^Full study names are listed in Table 1

### Data sources

The analytic model consists of two main inputs: enrollment data and financial data. Sites’ actual enrollment contributions, total enrollment in the study, and the length of time the study was open to enrollment were used to calculate annual enrollment rates. These rates were calculated at the site level and at the aggregate level in a stepwise fashion, or in order from highest enrollment rate to lowest enrollment rate, adding one site’s contribution to the overall annual enrollment at a time. The aggregate annual enrollments were then used to calculate how long the study would have had to stay open to reach the total enrollment target, again using a stepwise approach. The more sites that are added, the faster the enrollment target is reached.

Within the financial inputs, there are 3 direct cost components: (1) site costs, (2) study costs, and (3) overhead costs. Site costs consist of administrative start-up costs and single IRB costs. Study costs are the performance-based payments made to participating sites for enrollment and follow-up. Overhead costs are costs of METRC Coordinating Center personnel and general costs, e.g., printing, shipping, and general supplies.

The inputs for each of these main cost components were derived from METRC’s experience except for the single IRB, which none of the included studies actually used. However, now that single IRB use is compulsory, these costs were critical to making the model relevant and applicable to future studies. The single IRB costs used in the model are based on the Johns Hopkins School of Medicine Single IRB (JHM sIRB) fee schedule. Currently, the JHM sIRB fees are very representative of the market costs for both academic institution-based single IRBs and commercial IRBs; however, this may change over time as new providers come to market.

Of the 12 included studies, 7 were funded as part of consortium grants and 5 were funded as independent studies. In its early years, METRC used different financial management models for different types of funding. However, METRC’s current approach is consistent across funding mechanisms; sites are paid based on enrollment and follow-up performance with some funds given early on to get the study up and running. For this reason, all costs were configured according to the current, performance-based payment financial management model.

### Cost models

Three plausible cost models were used to determine the sensitivity of the model to changes in overhead costs, the most variable component of the study budget. For METRC studies, the bulk of overhead costs are associated with the METRC Coordinating Center (MCC) costs, i.e., the cost to run a trial through the consortium and not independently. MCC costs are determined based on a formula approved by METRC governance. They correspond with the total grant award amount which in turn corresponds with study complexity and the effort needed to implement studies successfully. As award amounts and complexity go up, so do MCC costs.

Each METRC study is handled by a principal investigator, key co-investigator(s), biostatistician, data analyst, project director, and study manager who together conduct all aspects of protocol development, study implementation, monitoring, data analysis, and preparation of primary and secondary results reports. Finance, administrative, and IT staff who are centralized within the MCC are responsible for handling budgets and contracts, and for building and maintaining study databases. Table 4 shows the level of salary support for these MCC personnel during the first, interim, and final years of a $1M, $3M, and $10M 4-year study. Total MCC costs are given for each cost model; the aggregate costs are drawn from real salaries and fringe of the noted personnel. The cost models are ordered from the lowest to highest cost for ease of interpretation, but cost model 2 is the main model within this study as it is most representative of a typical METRC study (~ $3M in funding) and most approximate to the actual MCC costs of the included studies (around $362,736). It is important to emphasize that cost models 1 and 3 (~ $1M and ~ $10M in funding) are also realistic overhead cost scenarios, albeit not approximate to the actual costs of the included studies.

**Table 4:** METRC Coordinating Center personnel salary support and total funding amounts by cost model

Personnel	Cost model 1 ($1M funding)			Cost model 2 ($3M funding)			Cost model 3 ($10M funding)		
	4-year MCC^a^ costs: $133,056			4-year MCC costs: $362,736			4-year MCC costs: $1,132,560		
	First	Interim	Final	First	Interim	Final	First	Interim	Final
MCC PI	5%	2%	5%	10%	5%	10%	25%	15%	25%
Biostatistician	5%	2%	5%	10%	5%	10%	25%	15%	25%
Co-investigators	1%	1%	1%	2%	2%	2%	10%	5%	10%
Project director	10%	5%	5%	20%	15%	15%	50%	50%	50%
Study manager	5%	8%	5%	15%	20%	20%	50%	50%	50%
Data analyst	2%	2%	5%	10%	15%	15%	50%	50%	50%
IT staff	2%	1%	2%	5%	10%	5%	30%	30%	30%
Finance/admin	1%	1%	1%	5%	2%	5%	15%	15%	25%

^a^Major Extremity Trauma Research Consortium (METRC) Coordinating Center (MCC)

## Results

**Fig. 2:**
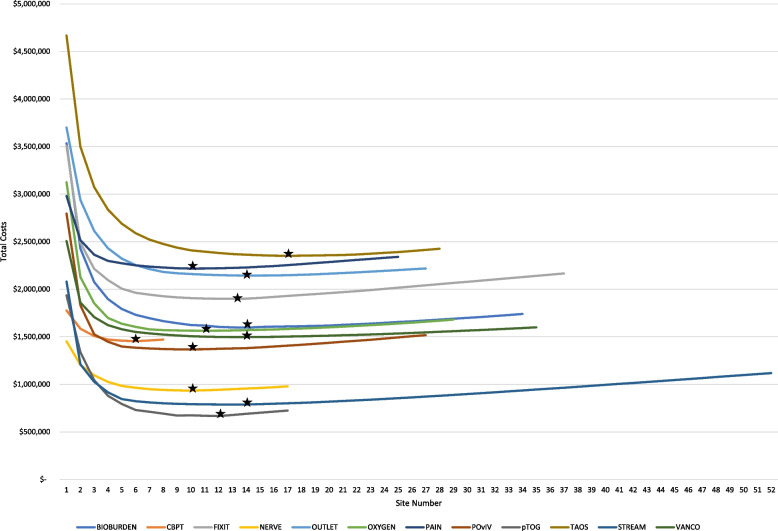
Total cost curves of included studies by number of participating sites*. *The stars represent the optimal number of sites. Full study names are listed in Table 1

A graph was made to depict the results of the main cost model, cost model 2 (Fig. 2). This graph represents the cost curve for each included study where the number of sites is reflected on the *X* axis and total costs on the *Y* axis. There is a considerable drop in total costs as the initial sites are added to the study. With only a handful of sites, the time it would take to reach enrollment targets would result in costs that are much greater than actual award amounts. Multicenter trials improve external validity and allow you to achieve the sample size needed to detect the effect of an intervention, where a sample of that size could never be reached by a single-center trial [26].

The figure shows the points along each cost curve at which the total costs bottom out, and after which they start to rise again (Fig. 2). The optimal number of sites is the point at which total costs are the lowest; this point for each study is marked with a black star. The reason total costs start to increase after hitting a low point is because, in terms of percentage of total enrollment, sites’ contributions are heavily right-skewed even though all sites cost the same to the study. As low enrolling sites are added to the study, the corresponding additional site costs outsize the gains made in time to study completion. In addition to confirming our hypothesis that it is possible to determine the optimal number of sites, the model and this graph also reveal this important relationship between site enrollment performance and total study costs.

The results of the sensitivity analysis are shown in Table 5. Ordered from the lowest cost model (cost model 1) to the highest cost model (cost model 3), the results show that as the fixed overhead cost increases so does the optimal number of participating sites. There are just two exceptions to this—the PhBMP-2 versus Autograft for Critical Size Tibial Defects: A Multicenter Randomized Trial (pTOG) and Improving Recovery After Orthopedic Trauma: Cognitive-Behavioral Therapy Based Physical Therapy (CBPT) studies. For these studies, the optimal number of sites is the same for cost models 2 and 3 for two reasons. First, pTOG and CBPT had fewer participating sites than the other ten studies and the model, which is based on real sites and real enrollment data cannot simulate beyond the actual number of sites that participated in the study. The second reason is that the lowest enrolling sites enrolled very few patients despite receiving approval to enroll.

**Table 5:** Optimal number of sites and total study costs by METRC Coordinating Center costs model

		Cost model 1			Cost model 2			Cost model 3		
MCC costs ^a^		Low: $133,056			Intermediate: $362,736			High: $1,132,560		
Study^b^	Actual number of sites used	Optimal number of sites	Years	Total costs	Optimal number of sites	Years	Total costs	Optimal number of sites	Years	Total costs
BIOBURDEN	34	10	6.33	$1,262,405	14	5.53	$1,596,196	20	5.04	$2,569,073
CBPT	9	4	2.08	$1,349,289	6	1.69	$1,453,665	6	1.69	$1,821,734
FIXIT	37	6	8.15	$1,499,032	13	6.48	$1,899,148	15	6.33	$3,083,458
NERVE	17	6	4.17	$724,495	10	3.89	$934,055	15	3.14	$1,582,993
OUTLET	27	8	4.91	$1,900,871	14	3.80	$2,143,628	20	3.36	$2,821,824
OXYGEN	29	7	5.96	$1,237,749	11	5.29	$1,563,808	20	4.60	$2,491,668
PAIN	25	6	5.23	$1,950,631	10	4.40	$2,217,465	14	4.06	$3,008,738
POvIV	28	5	7.63	$962,856	10	6.60	$1,368,154	15	6.16	$2,543,342
pTOG	17	9	5.59	$352,652	12	5.08	$666,424	12	5.08	$1,623,751
STREAM	52	6	5.22	$524,679	13	4.00	$787,874	20	3.54	$1,508,087
TAOS	28	10	7.67	$1,981,525	17	6.13	$2,351,391	23	5.67	$3,446,207
VANCO	35	7	3.94	$1,309,569	14	2.94	$1,497,082	23	2.54	$2,040,911

^a^Total Major Extremity Trauma Research Consortium (METRC) Coordinating Center (MCC) costs are presented as total direct costs for the Coordinating Center for a 4-year study, as calculated in Table 4

^b^Full study names are listed in Table 1

**Fig. 3:**
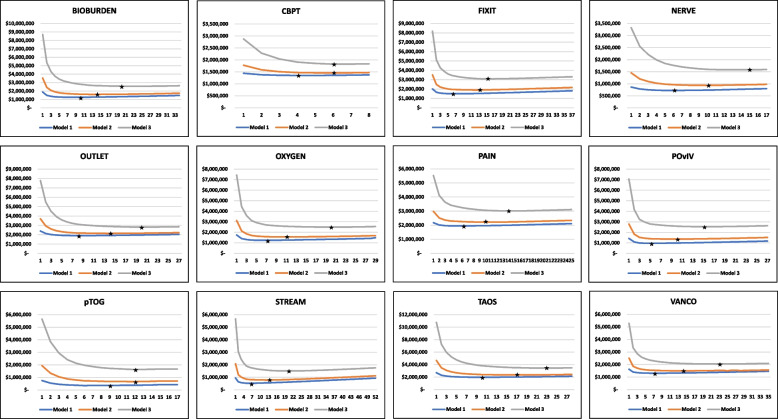
Total cost curves by cost model*. *The stars represent the optimal number of sites. Full study names are listed in Table 1

Each individual study is depicted in Fig. 3 with the addition of the low and high cost models. It is easy to see, again except for the pTOG and CBPT studies, that the higher the total fixed costs, the higher the optimal number of sites. Worth noting, as it cannot be seen in these figures, is that with the incremental addition of MCC costs, and correspondingly the addition of sites, the study does “save” some time because enrollment targets can be reached more quickly.

**Fig. 4:**
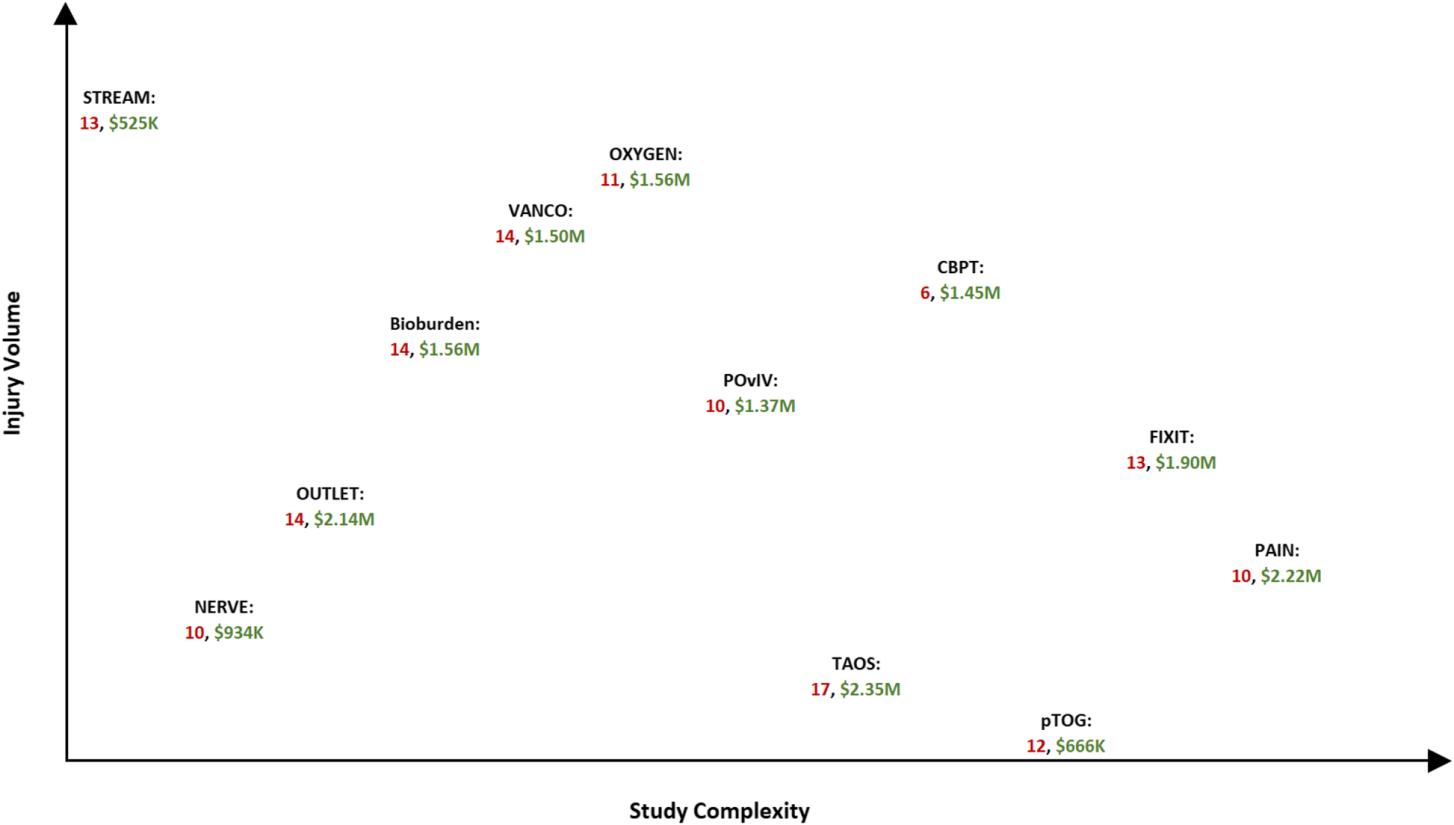
Optimal number of sites determined using cost model 2*. *Intermediate Major Extremity Trauma Research Consortium (METRC) Coordinating Center (MCC) costs. Full study names are listed in Table 1

Figure 4 shows where each of the included studies exists along the injury volume and study complexity continua, but additionally notes the optimal number of sites, in red, and corresponding total costs, in green. While no clear relationships emerge, there is a slight one between the optimal number of sites and the study characteristics of injury rarity and design complexity. The number of optimal sites is higher in two scenarios. The first is when injury volume is high and study complexity is low. Studies with higher injury volumes typically correspond with higher event or outcome rates. These studies require large sample sizes to detect intervention effects and more sites are needed to reach the large samples. Secondly and conversely, when injury volume is low, typically so is the event or outcome rate. For these studies, even though the sample size is small, it is hard to achieve and therefore requires more sites.

## Discussion

This study demonstrated that one can determine the number of optimal sites to have in a multicenter clinical trial when key study characteristics are known and when study costs and site enrollment performance are predictable. For the studies included, any participating site beyond the optimal number of sites could be considered excess, and there are quantifiable excess costs associated with those sites. Fortunately, while there is a clear optimal number of sites, the marginal cost of adding excess sites was on average just 7% of the overall study budget for these studies. This excess spending alone is likely not large enough to determine study success or failure and site startup costs are often more significant than site maintenance costs. However, under the current single IRB mandate, there are annual regulatory fees that must be paid for each participating site, and consortiums end up investing a lot of resources (i.e., person-hours) into identifying and overcoming site-specific barriers at sites that are underperforming. In the context of limited funding, it is critical to identify all sources of non-essential spending so that those resources may be redirected to other programmatic activities or decisions which contribute to the study’s success. For example, investigators could increase performance-based payments for sites, increase sample sizes to improve power, or increase impact through additional outcome data collection.

This study also demonstrated that while both site costs and infrastructure costs are key cost drivers of the study budget, the most significant of the two is highly contextual and largely driven by the level of overhead needed to complete the studies successfully. As overhead costs increase, so do the optimal number of sites as it becomes advantageous to complete the study as quickly as possible. For multicenter studies conducted within limited research networks, or for which participating sites make minimal enrollment contributions, high-cost studies with significant overhead burden may be less likely to succeed.

The model is particularly useful when site enrollment performance can be predicted. Extensive and mature research networks may be well-positioned to predict the three to five top enrolling sites and the sites which will contribute the fewest patients. Less predictable are the sites with mid-range enrollment contributions. These sites may be more vulnerable to changes within study teams or institutional policies and their enrollment performance rank, relative to all other sites in the network, is more likely to fluctuate within that mid-range. While enrollment performance is less clearly predictable for these sites, their enrollment contributions are essential for meeting requisite sample sizes within real-world funding periods.

A limitation of this study’s model is that its usefulness is a function of how well you can predict the distribution of enrollment contributions from your potential sites. Trials staff are often unreliable at predicting recruitment volume [27], but METRC is in a unique position where within consortium data from multiple analogous trials ensures predictability of enrollment. With more than 10 years of site enrollment data across many studies of varying size and complexity, most of the time we can confidently predict which sites will be the top enrollers and the bottom enrollers in a new study, especially if it is characteristically similar to an earlier study. Recruitment predictability makes the model highly useful for METRC.

The findings suggest that it is within the middle-of-the-pack group of sites that the line between optimal and excess sites is drawn. One area for future research is to determine predictive ability, that is, how well past enrollment performance predicts future enrollment performance. In the absence of confident enrollment predictions, trialists using our model should plan on a buffer—a few more sites than the model would suggest having participate [28]. Early monitoring can then detect low-performing sites, and these sites can be dropped, bringing the final number of sites in the study more proximal to the optimal number as determined by the model.

## Conclusions

When key study characteristics are known and study costs and site enrollment performance are predictable, it is possible to determine the number of optimal sites to have in a multicenter clinical trial. Our model is just one way to leverage the administrative and financial data that accumulate in a research consortium or network setting to build and manage organizational knowledge assets. While it cannot reveal the absolute truth of how many sites are optimal, it does provide information which is more approximate to the truth than best guesses made in the absence of data.

### Supplementary Information

**Additional file 1.**

## Data Availability

The datasets used and/or analyzed during the current study are available from the corresponding author upon reasonable request.

## Abbreviations

CBPT: Improving recovery after orthopaedic trauma: cognitive-behavioral therapy based physical therapy
IRB: Institutional review board
JHM sIRB: Johns hopkins school of medicine single IRB
METRC: Major extremity trauma research consortium
MCC: METRC coordinating center
pTOG: PhBMP-2 versus autograft for critical size tibial defects: a multicenter randomized trial
